# Natural Diterpenoid Oridonin Ameliorates Experimental Autoimmune Neuritis by Promoting Anti-inflammatory Macrophages Through Blocking Notch Pathway

**DOI:** 10.3389/fnins.2019.00272

**Published:** 2019-04-02

**Authors:** Lu Xu, Lei Li, Chen-Yang Zhang, Hermann Schluesener, Zhi-Yuan Zhang

**Affiliations:** ^1^Department of Pathology, Nanjing Medical University, Nanjing, China; ^2^Division of Immunopathology of the Nervous System, Institute of Pathology and Neuropathology, University of Tübingen, Tübingen, Germany; ^3^Department of Neurology, Sir Run Run Hospital, Nanjing Medical University, Nanjing, China

**Keywords:** Guillain-Barré syndrome, experimental autoimmune neuritis, Oridonin, anti-inflammatory macrophage, Notch pathway

## Abstract

The diterpenoid compound, Oridonin, extracted from the Chinese herb, *Rabdosia rubescens*, possesses multiple biological activities and properties. Oridonin exhibited efficient anti-inflammatory activity by inducing a switch in macrophage polarization to the anti-inflammatory phenotype through inhibition of the Notch pathway in our *in vitro* study; therefore, its potential therapeutic effects were further investigated in the animal model of human Guillain-Barré syndrome (GBS) and other polyneuropathies – experimental autoimmune neuritis (EAN). Either preventive or therapeutic treatments with Oridonin greatly attenuated disease peak severity, suppressed paraparesis, shortened disease duration, and even delayed EAN onset. Progression of neuropathic pain, demyelination, inflammatory cellular accumulations, and inflammatory cytokines in peripheral nerves were significantly attenuated. Meanwhile, accumulation of immune cells in the spinal roots and microglial activation in the lumbar spinal cord were also reduced. Interestingly, Oridonin treatment significantly increased the proportion of anti-inflammatory macrophages and made them locally dominant among all infiltrated macrophages in the peripheral nerves. The down-regulation of local Notch pathway proteins, together with our *in vitro* results indicated their possible involvement. Taken together, our results demonstrated that Oridonin effectively suppressed EAN by attenuating local inflammatory reaction and increasing the proportion of immune regulating macrophages in the peripheral nerves, possibly through blockage of the Notch pathway, which suggests Oridonin as a potential therapeutic candidate for human GBS and neuropathies.

## Introduction

Guillain-Barré syndrome (GBS) is an immune-mediated polyneuropathy; the world’s leading cause of acute autoimmune neuromuscular paralysis, caused by an autoimmune attack on the peripheral nervous system (PNS) (Willison, 2005; Kuwabara and Yuki, 2013). Existing treatments, including supportive managements, like advanced intensive care and assisted respiration, as well as some active treatments such as plasma exchange and intravenous immunoglobulin, are not satisfactory (Hughes, 2002). Not all GBS patients respond to these treatments, and even if they do, they still do not recover fully; and continuing weakness, motor units deprivation, and the problem of fatigue continues to be detected (Winer, 2001; Kieseier et al., 2004). Therefore, more effective, and convenient therapeutic approaches for the inflammatory peripheral demyelinating disease are needed. Experimental autoimmune neuritis (EAN), an autoantigen-specific, T cell-mediated animal model for demyelinating inflammatory diseases of the PNS, is broadly accepted and used to study pathogenesis, pathological changes, and potential therapeutic approaches for human polyneuropathies like GBS, by mirroring many of their neurological, electrophysiological, immunological, and pathological features (Hughes and Cornblath, 2005).

Experimental autoimmune neuritis is actively induced by immunization with autoantigen-like purified myelin or P2 peptides. Following the breakdown of the blood-nerve barrier, reactivated T cells and macrophages are abundantly infiltrated and accumulated in the PNS, which results in a robust local inflammation, demyelination, and axon degeneration, and all of these are crucial for the progression of EAN (Kieseier et al., 2004; Zhang et al., 2008a). Modulating or controlling the activation of these immune cells is, thus, considered an important therapeutic strategy for EAN and human neuropathies. As the main infiltrating cell population and the major effector cell present during EAN progression, macrophages are responsible for most neuropathological changes due to their antigen-presenting property and direct phagocytosis of axon myelin (Kiefer et al., 2001; Maurer et al., 2002). Infiltrating macrophages also cause tissue damage by secretion of inflammatory cytokines such as interleukin-1β (IL-1β) and tumor necrosis factor (TNF)-α, and these are responsible for demyelination and axon loss as well (Kiefer et al., 2001). Inhibition of macrophage activity or their depletion was proved to prevent EAN, as observed through all the clinical, electrophysiological, and histological changes (Craggs et al., 1984). However, recruited macrophages can be broadly divided into two particular phenotypes: classically activated macrophages that promote inflammatory reaction and tissue damage and alternatively activated macrophages with anti-inflammatory activity and tissue-repairing effects (Mosser and Edwards, 2008; Ricardo et al., 2008). Pro-inflammatory macrophages execute host-defense against infection, but also cause inflammatory tissue damage and lead to the high expression of pro-inflammatory cytokines such as IL-12, IL-23, and TNF-α and inducible nitric oxide synthase (iNOS). Anti-inflammatory macrophages are considered to control inflammatory reactions and are responsible for tissue repair; they are characterized by the high expression of anti-inflammatory molecules such as IL-10 and arginase-I (Mosser and Edwards, 2008; Ricardo et al., 2008). It has been previously reported that a phenotypic switch of macrophage polarization to the anti-inflammatory state can attenuate pathological progression and even determine the outcome of EAN (Zhang Z. Y. et al., 2010; Han et al., 2016a,b).

Plant-derived compounds have gained increased attention as nutraceutical treatments for various diseases, including cardiovascular diseases, mental disorders, cancers, and inflammatory diseases (Kumar, 2006). Oridonin, a natural diterpenoid compound extracted from a Chinese herb *Rabdosia rubescens* (Zhang Z. et al., 2010), exhibits numerous biological activities and effects, including oxygen free-radical clearing, anti-mutagenic, and anti-microbial activities, and is recently being studied intensively for promising anti-neoplastic activity (Ji et al., 2011, Jia et al., 2012). Moreover, its anti-inflammatory activities (He et al., 2018; Zhou et al., 2018), especially anti-neuroinflammatory and neuroprotective effects, have been reported recently in different *in vitro* and *in vivo* studies (Liu et al., 2007; Xu et al., 2009; Chen et al., 2012), which indicates its therapeutic potential in treating inflammatory disorders of the nervous system. As a widely used Traditional Chinese Medicine (TCM) and a main ingredient of many commonly available over-the-counter (OTC) herbal medicines in China, *R. rubescens* and its aqueous extract have shown excellent tolerance and oral bioavailability; therefore, the proven safety and reliability of the herb further ascertains its promising potential in clinical applications (Chen et al., 2009; Ma et al., 2011; Cheng et al., 2017).

Notch signaling is initiated by the Notch receptor-ligand binding (Mailhos et al., 2001). Typical signaling proteins such as Notch1, Jagged-2, and Hes-1 are usually tested for the activation or inhibition of Notch signaling, and were also investigated in our study. After Oridonin showed efficient anti-inflammatory activity and induced a phenotypic switch of macrophage polarization toward the anti-inflammatory state in our cell culture, the potential therapeutic effects of Oridonin were further investigated in the rat model of EAN, as we hypothesized that the possible effects might be connected to the regulation of immune cells, especially macrophages polarization.

## Materials and Methods

### Animals

Male Lewis rats (10–12 weeks, 230–250 g, Charles River, Sulzfeld, Germany) were housed under a 12 h light-12 h dark cycle with free access to food and water. The guideline EU Directive 2010/63/EU for animal experiments was followed and all animal procedures were approved by the local Administration District Official Committee. All efforts were made to minimize the number of animals and their suffering.

### Isolation of Primary Peritoneal Macrophages and Cell Culture

The rats were treated with 4 ml thioglycollate medium intraperitoneally. Seven days later, peritoneal cells were obtained by peritoneal lavage with 12 ml of ice-cold PBS (phosphate-buffered saline) containing 2% FBS (fetal bovine serum) (Invitrogen, Karlsruhe, Germany). FITC-conjugated anti-CD68 antibodies (Serotec, Oxford, United Kingdom) were used to confirm the purity of the macrophages and more than 90% of the peritoneal cells were macrophages.

Primary peritoneal macrophages and a common murine macrophage cell line, RAW 264.7, (Sigma-Aldrich, Munich, Germany) were both used to evaluate the effects of Oridonin on the inflammatory reaction and polarization of macrophages *in vitro*. Primary macrophages and RAW cells were maintained in complete RPMI 1640 media, from Gibco (Invitrogen, Karlsruhe, Germany), containing 10% FBS (Invitrogen, Karlsruhe, Germany), penicillin (100 U/ml), and streptomycin (100 U/ml). 10^5^ cells were seeded into 12-well plates. After 24 h of culture, lipopolysaccharide (LPS) (Sigma-Aldrich, Munich, Germany) was added in some wells and co-cultured for another 24 h; some groups were treated with Oridonin (0.25, 1, or 4 μM). A previous pharmacokinetic study (Xu et al., 2006) reported that the plasma concentrations of Oridonin after oral dosing of 20 mg/kg or 40 mg/kg in rats were between 0.1 and 0.35 μg/ml for over 10 h, which were equivalent to 0.25 to 0.95 μM. Our *in vitro* study, therefore, used working concentrations of 0.25, 1, and 4 μM. Further, some groups of cells were incubated for 48 h with 30 μM DAPT (Sigma-Aldrich, Munich, Germany), an inhibitor of the Notch signaling pathway. Thereafter, supernatants from different wells were collected and a standard Griess assay (Sigma-Aldrich, Munich, Germany) was performed to analyze the production of nitric oxide (NO). Total RNA was extracted from cells with the RNeasy Mini Kit (QIAGEN, Hilden, Germany) according to the manufacturer’s protocol. QuantiTect Reverse Transcription Kit (QIAGEN, Hilden, Germany) was utilized to reverse transcribe RNA (1 μg) into cDNA. Real-time PCR analysis was used to measure the mRNA expression. ELISA kits for IL-1β (Thermo Scientific, Waltham, MA, United States), TNF-α, and IL-10 (Bio legend Inc., San Diego, CA, United States) were used to detect the concentrations of the cytokines in macrophage culture supernatants.

### Transient Transfection

The Notch1-NICD (Notch intracellular domain) was generated using the following primers: sense 5′-CGCGGATCCATGCACCTGGATGCCGCTGACCTG-3′ and antisense 5′-ACGTCTAGACTYGAAGGCCTCCGGAATGCG-3′. The pair of primers were designed for Notch1-NICD gene sequences from the GenBank database, and the fragment of Notch1-NICD was cloned into the pcDNA3.1 vector (Invitrogen, Karlsruhe, Germany). Lipofectamine 2000 (Invitrogen, Karlsruhe, Germany) was used to conduct transfection based on the manufacturer’s protocols. The Real-time-PCR was conducted to monitor the transfection efficiency. RAW cells from the other comparable groups, including the control group, were all transiently transfected with a plasmid vehicle.

### EAN Induction and Oridonin Treatment

After dissolving in PBS at a concentration of 2 mg/ml, the synthetic neurogenic peptide, P2 57-81 (GeneScript Corporation, Scotch Plains, NJ, United States) was emulsified with 2 mg/ml *Mycobacterium tuberculosis* mixed within Complete Freund’s Adjuvant (CFA) (Sigma-Aldrich; Munich, Germany), and the final concentration of peptide in the emulsion was 1 mg/ml. Each rat was injected subcutaneously at the base of the tail with 100 μl emulsion containing 100 μg peptide.

Once a day, neurological signs of EAN were recorded and graded as follows: normal (score = 0), reduced tonus of tail (score = 1), impaired righting, limp tail (score = 2), absent righting (score = 3), gait ataxia (score = 4), mild paresis of the hind limbs (score = 5), moderate paraparesis (score = 6), severe paraparesis or paraplegia of the hind limbs (score = 7), tetraparesis (score = 8), moribund (score = 9), and death (score = 10).

Four rounds of Oridonin treatments were performed: two each of therapeutic and preventive treatments. EAN rats received daily gavages of Oridonin from Day 0 to Day 14 as the preventive treatment or from Day 9 to Day 14 as the therapeutic treatment (six rats/group); rats were prepared for tissue analysis on Day 15. In addition, another three groups of rats administered identical treatments as described were observed until they fully recovered to cover the entire spectrum of disease development and recovery. Oridonin, suspended in 1% carboxymethylcellulose (CMC) (Blanose^®^, Hercules-Aqualon, Düsseldorf, Germany), was fed by gavage once a day at doses of 7 or 20 mg/kg of body weight.

It was reported that 175 mg of Oridonin administered orally to humans, which is a common dosage prescribed for inflammatory conditions in TCM, was comparable to the dose of 13.5 mg/kg administered to rats (Xu et al., 2006). The oral dosing of 20 mg/kg was comparable to the treatments of lupus-like symptoms (Zhou et al., 2013) and neuroinflammation of cerebral amyloidosis (Zhang et al., 2013) in mice. A daily dose of 10 mg/kg of body weight administered by the intraperitoneal (i.p.) route was reported previously to treat animal models of rats (Li et al., 2018). A previous pharmacokinetic study of Oridonin indicated that the oral absolute bioavailability of Oridonin was rather low and it was approximately 1/2–1/3 that of i.p. treatments (Xu et al., 2006). The same pharmacokinetic study further reported that the plasma concentrations of Oridonin after oral dosing of 20 mg/kg or 40 mg/kg in rats were between 0.25 and 0.95 μM, which is comparable to the working concentrations used in our *in vitro* study. All these reports indicated that the oral dose of 20 mg/kg to EAN rats is comparable to the dosage reported in most published studies and for human treatments in TCM with the herb, *R. rubescens*. Additionally, we tested the dose of 7 mg/kg (1/3 of the 20 mg/kg dose) in the preventive treatment round to observe the dose-dependent effect.

### Mechanical Allodynia

Mechanical allodynia was defined by the reduction of the hind-paw withdrawal threshold (HWT), which was measured using a mechanical plantar test apparatus, namely, an automatic von Frey system (Ugo Basile, Milan, Italy). All rats were trained every day, starting six days before the first HWT measurement. HWT was examined between 10:00 and 14:00 every day from two days prior to immunization to 14 days post-immunization. Briefly, rats were habituated for 10 min and acclimated for another 10 min. The mechanical force (from 0 to 50 *g* over a period of 15 s) was exerted onto the middle of the hind-paws using a fine metal filament. Left and right hind-paws were measured 10 times each, one after the other. The forces under which the rats actively lifted their paws on their own initiative were recorded and mean values were calculated. All EAN rats for mechanical allodynia assays were re-grouped before immunization according to the measurement results of the training sessions to stratify the experimental groups.

### Immunohistochemistry

On Day 15, after deep anesthesia and intracardial perfusion with 4% paraformaldehyde at 4°C, the rats were prepared for analysis. To detect the infiltration of inflammatory cells and the levels of pathological changes in the PNS, sciatic nerves from both the legs and the lumbar spinal cord were removed quickly and fixed overnight in 4% paraformaldehyde at 4°C. Each specimen was cut into two equal long segments, embedded in paraffin, serially sectioned (3 μm), and mounted on slides.

Sections were dewaxed and boiled in a microwave oven (600 W) in citrate buffer (sodium citrate 2.1 g/L, pH 6) for 15 min. Methanol containing 1% H_2_O_2_ was used to inhibit endogenous peroxidase for 15 min. After blockage with 10% pig serum (Biochrom, Berlin, Germany), cross sections were incubated with monoclonal antibodies as follows: CD3 (1:50; Serotec, Oxford, United Kingdom) for T cells, OX22 (1:200; Serotec, Oxford, United Kingdom) for B lymphocytes, glial fibrillary acidic protein (GFAP) (1:500; Chemicon International, Temecula, CA, United States) for astrocytes. Activated macrophages/microglia and anti-inflammatory macrophages were detected by ED1 (a lysosomal membrane protein which recognizes CD68) (1:100; Serotec, Oxford, United Kingdom) and ED2 (1:100; Serotec, Oxford, United Kingdom), respectively. Biotinylated IgG F(ab)2 (DAKO, Hamburg, Germany) was used as secondary antibody fragment to visualize the antibodies binding to sections. A horseradish peroxidase-conjugated streptavidin complex (DAKO, Hamburg, Germany) and diaminobenzidine (DAB) substrate (Fluka, Neu-Ulm, Germany) was subsequently added to sections. Tissues were counterstained with Maier’s Hemalum in the end.

The ratio of immunoreactivity (IR) areas to sciatic nerve cross-section areas were calculated as described previously (Zhang et al., 2008a). Briefly, micro-images of staining were captured with a Nikon Cool-scope (Nikon, Düsseldorf, Germany); MetaMorph Offline 7.1 (Molecular Devices, Toronto, ON, Canada) was applied on the micro images to outline and analyze the cross sections of the sciatic nerves. In regions of interest, the percentages of specific IR were selected by color threshold segmentation and analyzed. For each staining, all parameters were fixed for all the images and no automatic adjustments were used. Results are shown as the arithmetic means of percentages of IR area to interest areas on cross-sections and standard errors of means (SEM).

Luxol Fast Blue (LFB) (Sigma-Aldrich; Munich, Germany) staining was used to visualize myelin. For quantification of the demyelination of impacted nerves, the software MetaMorph Offline 7.1 (Molecular Devices, Toronto, ON, Canada) was used to analyze the relative optical density of the nerves from all groups. Raw images of nerve cross sections were acquired at the same exposure level and converted to 8-bit gray scale files. For removing the image background, the mean density calculated from the threshold pixels.

Furthermore, between Oridonin-treated and control EAN rats, histological alterations were analyzed by a semi-quantitative method established previously (Zhang et al., 2008a). In short, cross-sections of middle levels of the left and right sciatic nerves from EAN rats were evaluated. All cross-sections were collected and perivascular areas were evaluated by two pathologists. The levels of pathological changes were semi-quantitatively graded as: normal perivascular area (0); mild inflammatory cellular infiltration in immediate proximity to the blood vessels (1); cellular infiltration and demyelination adjacent to the blood vessels (2); and cellular infiltration plus demyelination throughout the cross-sections (3). Results are shown as mean histological scores (Hartung et al., 1988).

### Tissue Preparation, RNA Isolation, Reverse Transcription, and Semi-Quantitative PCR

Experimental autoimmune neuritis rats were deeply anesthetized and intracardially perfused with 4°C PBS. The sciatic nerves, lumbar spinal cords, livers and inguinal lymph nodes were removed quickly and stored in liquid nitrogen immediately for RNA isolation. Trizol LS Reagent (Invitrogen, Karlsruhe, Germany) was applied to isolate total RNA and QuantiTect Reverse Transcription Kit (QIAGEN, Hilden, Germany) was used to reverse transcribe RNA to cDNA, The resulting cDNA was used to measure semi-quantitatively the expression of genes using SYBR green qPCR master mix according to the manufacturer’s protocol (BioRad, Hercules, CA, United States). Real-time measurements of gene expression were performed with an iCycler thermocycler system and iQ5 optical system software (BioRad, Hercules, CA, United States). All the results are presented as mRNA expression levels relative to the housekeeping gene β-actin.

### Flow Cytometric Analysis

Single-cell suspensions were prepared from the spleens taken from the rats on Day 15. RBCs were lysed and monocytes were suspended in 100 μL PBS containing 1% FBS. All cells were stained, according to the manufacturer’s protocol, with FITC-conjugated antibody against CD11b and anti-CD206-PE (eBioscience, San Diego, CA, United States), which is a specific anti-inflammatory marker. Data were analyzed using FlowJo Flow Cytometry Analysis Software ^1^.

### Western Blot Analysis

On Day 15, total proteins was extracted from the sciatic nerves of various groups of EAN rats. All samples were adjusted to an equal volume and content and separated electrophoretically on 12% SDS-PAGE gels. Then, proteins were transferred to PVDF membranes (Millipore, Billerica, MA, United States). After being blocked in Tris-buffered saline solution (TBS) containing 5% BSA for 2 h, membranes were incubated at 4°C overnight with primary antibodies: Notch1 (1:1000), Jagged-2 (1:500) (Cell Signaling Technology, Beverly, MA, United States), Hes-1 (1:500) (Millipore, Single Oak Drive, Temecula, CA, United States), and β-actin (1:500) (Sigma, St. Louis, MO, United States). The proteins were visualized by secondary antibodies with enhanced chemiluminescence. The signals of specific proteins were detected with a Gel Doc imager (Serial No. 721BR08844; Bio-Rad, Hercules, CA, United States) and reported as the fraction of control β-actin.

### Evaluation and Statistical Analysis

Differences between the neurologic scores and histological scores were evaluated with the Mann–Whitney *U* test. For paired and multiple group comparisons, Student’s *t*-test and one-way analysis of variance (ANOVA) were applied, respectively. The results are represented as means ± SEM. Statistical significance was defined as *p*-values < 0.05. Statistical analyses were performed using GraphPad Prism 5.0 (GraphPad Software Inc., San Diego, CA, United States). For the *in vitro* studies with cell cultures, graphs show mean ± SEM of triplicate wells and represent data from three independent experiments.

## Results

### Oridonin Inhibited Inflammatory Response and Induced Phenotypic Switch of Macrophage Polarization in Cell Culture

As the anti-inflammatory phenotype of macrophages can attenuate the inflammatory response and promote tissue repair, we first investigated whether Oridonin could inhibit the inflammatory response of the macrophages cell line, RAW, and the primary peritoneal macrophages by favoring a switch of macrophages from classically activated inflammatory into the anti-inflammatory phenotype *in vitro*.

**FIGURE 1:**
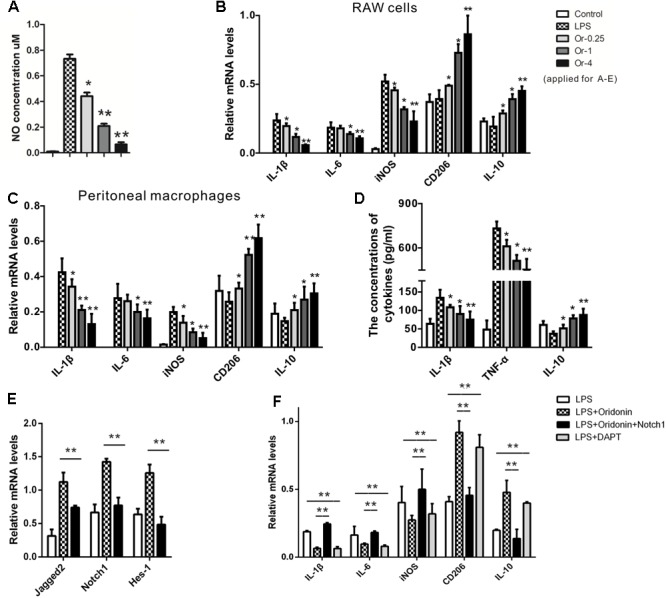
Effects of Oridonin on phenotypic switch of macrophage polarization and Notch pathway *in vitro*. *In vitro* cultured macrophage cells were stimulated by LPS, transfected with Notch1 or vehicle plasmid, and treated with Oridonin or DAPT. NO concentrations, proportions of PCR products relative to the house keeping β-actin and cytokine concentrations are presented as bar graphs. **(A)** Upon the induction of LPS, NO production in RAW cell culture was increased compared to the control. Further, NO concentrations were significantly reduced by Oridonin treatment, in a clear dose-dependent pattern. **(B,C)** In both RAW cells and primary macrophages, the RNA levels of IL-1β, IL-6 and iNOS, the markers of inflammatory macrophages, were significantly decreased by Oridonin treatment. On the contrary, CD206 and IL-10 mRNA levels, the markers of anti-inflammatory phenotype, were significantly increased. All these results also presented clear dose-dependent patterns. **(D)** ELISA assays showed that the concentrations of IL-1β and TNF-α were reduced by Oridonin treatment and the level of IL-10 was increased in primary macrophages, in a dose-dependent pattern as well. **(E)** mRNA levels of Notch pathway involved molecules Jagged-2, Notch1 and Hes-1 were remarkably inhibited by Oridonin treatment in RAW cells. **(F)** In RAW cells, increased mRNA levels of IL-1β, IL-6 and iNOS were induced by LPS. Their levels were decreased by Oridonin or DAPT, whereas the levels of CD206 and IL-10 were reversed. But the Oridonin’s effects were abolished by the transient transfection of Notch1. Cells from the other groups were all transiently transfected with vehicle plasmid. (Or-0.25, -1, -4 = Oridonin concentrations of 0.25, 1, and 4 μM. ^∗^*p* < 0.05, ^∗∗^*p* < 0.01 compared to their respective controls). Graphs show mean ± SEM of triplicate wells and represent data from three independent experiments.

Upon LPS induction, increased NO production (Figure 1A) and mRNA expression of characteristic inflammatory cytokines IL-1β, IL-6, and iNOS (Figure 1B) indicated a classically pro-inflammatory phenotype in both the RAW cells and the primary macrophages. Oridonin treatments significantly reduced the NO production (Figure 1A) and reduced mRNA levels of these inflammatory cytokines in dose-dependent patterns, suggesting a suppressed inflammatory reaction (Figure 1B,C). Further, mRNA levels of anti-inflammatory phenotype markers IL-10 and CD206 were significantly increased, also in dose-dependent patterns, indicating a switch in the phenotype of the macrophages to the anti-inflammatory phenotype (Figure 1B,C). Additional MTT assays excluded possible differences in cell viability among the different treatments (data not shown).

The expression levels of IL-1β, TNF-α, and IL-10 from the primary macrophages were detected with the ELISA assay. The productions of IL-1β and TNF-α were significantly reduced by Oridonin. Further, the macrophages treated with Oridonin released much more IL-10 than LPS induced cells (Figure 1D). Therefore, Oridonin showed efficient anti-inflammatory activity and induced a switch of macrophage polarization toward the anti-inflammatory phenotype, which suggested its further application in the inflammatory animal model of EAN.

### Oridonin Induced Phenotypic Switch in Macrophage Polarization by Blocking the Notch Signaling

We further investigated the possible mechanism behind the anti-inflammatory effects of Oridonin. The Notch pathway is a direct target of Oridonin, and Notch inhibition was reported to enhance monocyte differentiation into the anti-inflammatory (M2) phenotype (Dong et al., 2014; Singla et al., 2014, 2017; Xia et al., 2017). We, therefore, investigated whether Oridonin exerts these effects by affecting Notch signaling. In LPS-stimulated RAW cells, Oridonin treatment significantly reduced the mRNA levels of Jagged-2, Notch1, and Hes-1, all of which are involved in the Notch pathway (Figure 1E). Further, the effects of Oridonin were abolished by the Notch1 transient transfection. Cells from the other comparable groups, especially the control group, were all transient transfected with the plasmid vehicle. Simultaneously, the Notch inhibitor DAPT exhibited anti-inflammatory activity similar to that of Oridonin, the mRNA levels of pro-inflammatory cytokines, including IL-1β, IL-6, and iNOS were decreased, and the anti-inflammatory phenotype markers IL-10 and CD206 were significantly increased (Figure 1F). Taken together, our results suggest that Oridonin induced the phenotypic switch of LPS-stimulated macrophages from the pro-inflammatory phenotype to the anti-inflammatory phenotype by blocking the Notch signaling.

### Oridonin Treatment Suppressed EAN Neurological Progression and Accompanying Mechanical Allodynia

**FIGURE 2:**
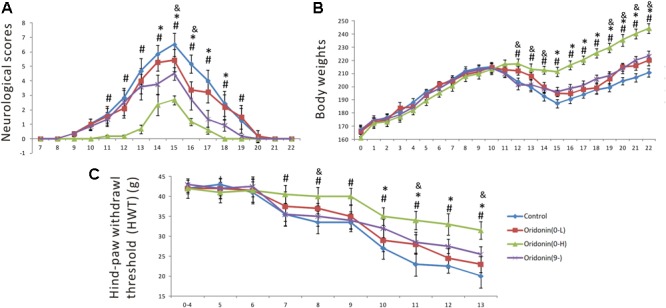
Preventive and therapeutic Oridonin treatments attenuated neurological disease severity, shortened disease duration and suppressed mechanical allodynia in EAN rats. EAN rats (*n* = 6) were orally administrated daily with Oridonin (7 or 20 mg/kg in CMC) or CMC alone (same volume), since Day 0 as preventive or since Day 9 (first neurological sign occurred) as therapeutic treatment. Neurological scores and body weights were recorded every day post-immunization. **(A)** Both preventive and therapeutic Oridonin treatments decreased neurological severity of EAN and shortened EAN duration. **(B)** Meanwhile, the reduction of body weights, which is also a characteristic sign of EAN, was significantly ameliorated by both therapeutic and preventive treatments with Oridonin. **(C)** The another characteristic neurological sign of EAN, mechanical allodynia, defined as reduction of HWT, was observed as early as Day 7 up to the end of the experiment (Day 13) in the control group. Either therapeutic or preventive treatment with Oridonin at high or low doses significantly restored the reductions. Significant differences of HWT among control, Oridonin therapeutic and preventive groups were confirmed. Finally, repeated ANOVA assays indicated significant difference between all the three groups, in neurological scores, body weight time courses and HWT. ^∗^*p* < 0.05 comparison of the preventive treatment group to the control group, ^&^*p* < 0.05 comparison of the preventive treatment group at 7 mg/kg to the control group, ^#^*p* < 0.05 comparison of the therapeutic treatment group to the control group, *n* = 6.

Experimental autoimmune neuritis was induced by subcutaneous immunization of Lewis rats with 100 μg of synthetic neurogenic P2 peptide. Oridonin or CMC (the control group) was given by daily gastric gavage starting at Day 0 as a preventive or on Day 9 (when the first neurological sign occurred) as a therapeutic treatment. In the control group, the first neurologic signs were established on Day 9 (0.33 ± 0.17); the neurologic severity represented by the scores peaked on Day 15 (6.3 ± 0.8), receded thereafter, and disappeared by Day 21 (0 ± 0). In comparison, preventive treatment of Oridonin at the dose of 20 mg/kg, initiated since Day 0, effectively delayed EAN onset (the first neurological sign occurred on Day 11), significantly reduced the severity at disease peak (2.7 ± 0.3, *p < 0.05*, compared to the control group), and shortened disease duration (until Day 18). Moreover, therapeutic treatment with the dose of 20 mg/kg, started after the first neurological sign was seen, also significantly attenuated the disease peak (4.5 ± 0.4, *p < 0.05*, compared to the control group) and lead to an earlier recovery from EAN (until Day 20) (Figure 2A). Both the therapeutic and preventive treatments with Oridonin significantly ameliorated the reduction of body weight, which is also a characteristic sign of EAN (Figure 2B). Additionally, a lower dose of 7 mg/kg also showed therapeutic, but significantly reduced, effects in the preventive treatment. Further, repeated ANOVA assays indicated a significant difference between all the four groups, both in the neurological scores and body weight time courses.

Mechanical allodynia is a characteristic neurological sign of both human neuropathies and animal EAN. For the EAN rats, it can be recorded as a significant reduction of the hind-paw withdrawal threshold (HWT) in comparison to individual baselines (average HWT of the first four days after immunization). Mechanical allodynia was established on Day 7 except in the group treated with Oridonin at the dose of 20 mg/kg from Day 0 (Figure 2C). In the control group, HWT decreased persistently and gradually, presenting a steady progression of mechanical allodynia. Meanwhile, in the therapeutic treatment group (since Day 9), reduction of HWT was steady, like in the control group until Day 9, but was significantly slower after that. As shown in Figure 2C, HWT values detected between the therapeutic treatment and control groups were significantly different since Day 10, one day after the first treatment, and the differences could be seen till Day 13, the end of the observation period (when the EAN rats in the control groups could not lift their paws anymore). In the group treated prophylactically with Oridonin (20 mg/kg) since Day 0, a significant reduction could not be seen until Day 10; the HWT decreased slowly after that, but the reduction was always a little less than in the other two groups. The additional group administered the preventive treatment with the lower dose of 7 mg/kg also showed protective, but significantly reduced, effects in this test. Our results indicate that Oridonin treatment significantly suppressed and slowed the progression of mechanical allodynia in the EAN rats.

### Oridonin Treatment Ameliorated Pathological Changes of EAN by Reducing Infiltration of Inflammatory Cells and Inflammation-Related Cytokines

**FIGURE 3:**
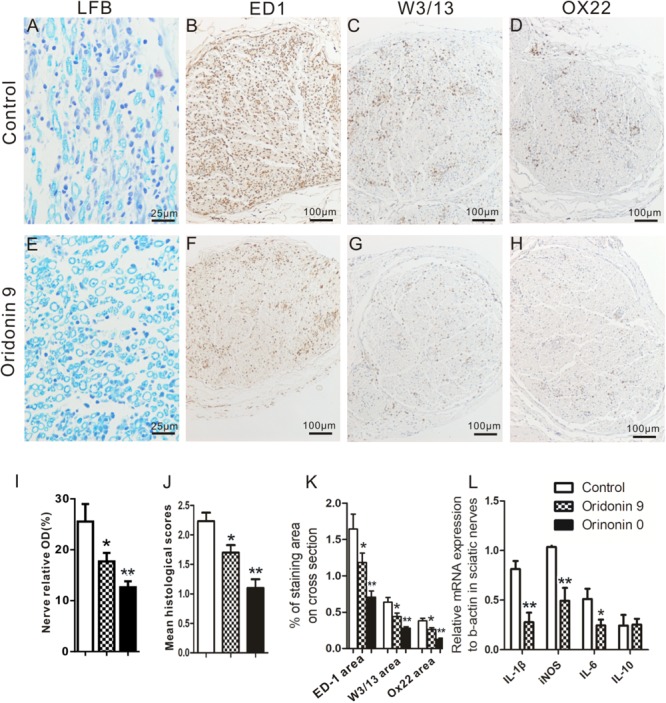
Oridonin treatment attenuated local demyelination, inflammatory cell infiltration and inflammatory cytokines in sciatic nerves of EAN rats. Following preventive or therapeutic treatments, rats were sacrificed on Day 15 and sciatic nerves were taken for LFB and immunohistochemical staining or semi-quantitative PCR. Representative microphotos of LFB and IHC staining for control **(A–D)** or Oridonin treated **(E–H)** EAN rats are shown. Quantification of demyelination, mean histological scores and percentages of immune cells marker IR on cross sections were calculated as described in Materials and Methods. Preventive or therapeutic Oridonin treatments significantly reduced the demyelination levels; mean histological scores; percentages of ED1 (macrophages), W3/13 (pan-T cells) and OX22 (B cells) IR in sciatic nerves, compared to the control EAN rats **(I–K)**. **(L)** mRNA levels of IL-1β, IL-6, iNOS and IL-10 in sciatic nerves of these EAN rats was analyzed by real-time PCR. Therapeutic Oridonin treatment significantly reduced mRNA levels of IL-1β, IL-6 and iNOS in sciatic nerves, but did not change the mRNA level of IL-10 significantly. ^∗^*p* < 0.05, ^∗∗^*p* < 0.01 compared to the control group, *n* = 6.

Following therapeutic or preventive treatments with CMC or Oridonin, EAN rats were prepared for analysis at the peak of the EAN progression, namely on Day 15, and sciatic nerves were collected for further histological analysis (*n* = 6). LFB staining and quantification were first performed to investigate nerve demyelination and local infiltration of immune cells. Severe perivascular demyelination and massive infiltration of inflammatory immune cells were observed in the control group. Oridonin treatment significantly attenuated the degree of demyelination and the severity of cellular infiltration (Figure 3A,E,I). These pathological changes were evaluated, scored, and compared between treatment and control groups. As shown in the Figure 3J, in comparison to the control group (2.23 ± 0.16), both Oridonin preventive- (Oridonin 0) and therapeutic treatments (Oridonin 9) significantly decreased the mean histological scores of the sciatic nerves (1.14 ± 0.08 and 1.68 ± 0.13, respectively, *p* < *0.05*).

Further immunohistochemical analysis showed significant reduction in the local aggregation of various infiltrated cellular populations following the Oridonin treatments. In the control group, massive infiltration of macrophages (ED1^+^), pan-T cells (W3/13^+^), and B cells (OX22^+^) were observed and the macrophages were dominant in the cross-sections (Figure 3B–D). These infiltrated cells were not uniformly distributed in the cross-sections but were more concentrated around vessels and in the perineurium; some of them were also seen in the endoneurium. Both preventive and therapeutic Oridonin treatments significantly attenuated infiltration of all these cell types *(p < 0.05*) but did not alter their distribution pattern (Figure 3F–H,K).

In addition, mRNA levels of inflammatory cytokines in sciatic nerves, including those of IL-1β, iNOS, and IL-6, which are crucial for inflammatory progression in EAN, were further analyzed by real-time PCR. As shown in Figure 3L, the mRNA levels were significantly lower in the Oridonin-treated group than in the control group. However, the IL-10 mRNA level was not significantly changed, probably due to the dramatically reduced macrophage number in the local region.

### Oridonin Treatment Decreased Inflammatory Cell Accumulation in Spinal Roots and Microglial Activation in the Spinal Cord

**FIGURE 4:**
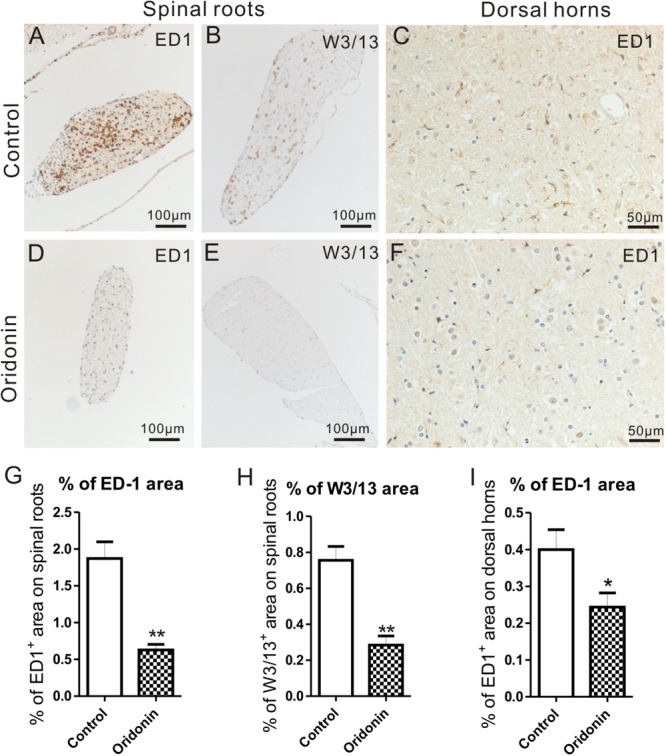
Oridonin treatments attenuated cellular infiltration in spinal roots and inflammatory microglial activation in lumbar spinal cords of EAN rats. EAN Rats were preventively treated with Oridonin and sacrificed on Day 15, spinal cords were taken for immunohistochemical staining and analyzed, especially focusing on lumbar sections. Significant accumulation of ED1^+^ and W3/13^+^ cells was mainly observed in dorsal roots of our EAN rats **(A,B)**. Similar to sciatic nerves, they were mainly detected to concentrate around vessels in perineurium and endoneurium. Following the Oridonin treatment, their accumulations were significantly reduced **(D,E,G,H)**. The expression of ED1 in lumbar spinal cords was further analyzed. In lumbar spinal cords of EAN rats, significantly increased ED1 expression could be observed in the control group **(C)**. ED1 IR was mainly detected in gray matter, particularly in the superficial layers of dorsal horns. The density of ED1 IR in dorsal horns was then investigated, and it was significantly decreased by Oridonin treatment **(F)**, compared to the control group **(I)**. ^∗^*p* < 0.05, ^∗∗^*p* < 0.01 compared to the control group, *n* = 6.

In EAN rats, significant accumulation of ED1^+^ and W3/13^+^ cells were observed in the dorsal and ventral roots. Like the sciatic nerves, they mainly accumulated around vessels in the endoneurium and perineurium. In comparison, macrophages and T cells were rarely seen in the dorsal and ventral roots of the spinal cord in naive animals and with a lower cell density than in sciatic nerves, as reported in our previous studies (Zhang et al., 2009). Following the preventive Oridonin treatment, their accumulations were significantly reduced, for ED1: 1.9 ± 0.3% in the control group and 0.6 ± 0.1% in the treatment group (*p < 0.05*) (Figure 4A,D,G); for W3/13: 0.8 ± 0.1% in the control group and 0.3 ± 0.1% in the treatment group (*p < 0.05*) (Figure 4B,E,H).

ED1 is also considered the microglial activation marker in CNS and its expression in the spinal cord was further analyzed. According to our previously published data (Zhang et al., 2009), only a few microglial cells could be observed in the spinal cord of naive rats. In our EAN rats, however, obvious ED1 IR could be seen, mostly in the gray matter of the lumbar spinal cord, especially in the superficial layers of the dorsal horns. They accumulated near large neurons, particularly in the control group. They exhibited an activated hypertrophic morphology, which was characterized by thicker, shorter, and less branched processes; and enlarged, darkened soma. In the Oridonin group, however, fewer ED1 IR and ED1^+^ cells with relatively smaller soma were observed (Figure 4C,F). The density of ED1 IR was then analyzed in the dorsal horns, where ED1^+^ cells were relatively more abundant. The quantity of ED1 IR was significantly lower in the Oridonin preventive treatment group than in the control group (0.40 ± 0.04% for control, 0.24 ± 0.03% for treatment, *p < 0.05*) (Figure 4I).

### Oridonin Increased the Proportion of Anti-inflammatory Macrophages in EAN Rats

**FIGURE 5:**
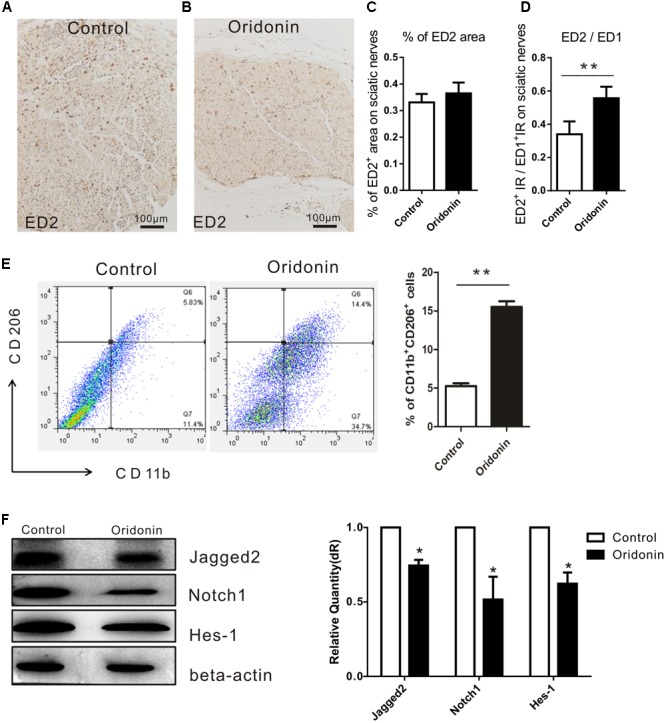
Oridonin increased proportion of anti-inflammatory macrophages in EAN rats through suppression of Notch pathway. Rats were treated therapeutically with Oridonin and sacrificed on Day 15; sciatic nerves were taken for immunohistochemical staining. By staining, anti-inflammatory macrophages were identified by expression of CD163, which is detected by ED2 antibody. Representative microphotos show ED2^+^ cells in sciatic nerves of EAN rats treated by Oridonin **(B)** in comparison to the control **(A)**. In the control group, much more infiltrates could be observed, but CD163 expression was not significantly different from the treatment group, where much less accumulated immune cells could be seen. **(C,D)**: The bar graphs show no difference in CD163 IR levels between control and Oridonin groups, but the proportions of ED2^+^ over ED1^+^ IR areas in sciatic nerves were significantly increased in comparison to controls. Further, the frequency of CD206^+^CD11b^+^ cells from spleen were tested by flow cytometry. **(E)** Spleen mononuclear cells (MNCs) were isolated from the control and Oridonin treated EAN rats at day 15 post-immunization and examined by flow cytometry. Percentage of anti-inflammatory macrophages (CD206^+^CD11b^+^ cells) in spleen MNCs was significantly increased in Oridonin treated rats compared to the control group. **(F)** On day 15, sciatic nerves of each rat were harvested for Western blotting. Relative expression of Jagged-2, Notch1 and Hes-1 in Oridonin-treated groups was significantly lower compared to the control group. ^∗^*p* < 0.05, ^∗∗^*p* < 0.01 compared to the control group, *n* = 6.

A significantly increased expression of CD163 is characteristically observed on an anti-inflammatory activated phenotype of macrophages (Moestrup and Moller, 2004; Badylak et al., 2008). ED2 antibody against CD163 was therefore used to detect these macrophages in the sciatic nerves of our EAN rats (Figure 5A,B). Even though the total numbers of accumulated macrophages were dramatically reduced (ED1^+^ cells, Figure 3B,F), CD163/ED2 IR (0.36 ± 0.04%) in the sciatic nerves of the Oridonin group was slightly increased (0.33 ± 0.03%, *p* > 0.05, compared to the control group, Figure 5C). Moreover, the proportion of CD163 IR among total macrophages (ED1^+^ cells) was significantly increased following the treatment (36% for the control and 57% for the Oridonin group, Figure 5D), indicating a switch of the local macrophage population from classically activated inflammatory into the anti-inflammatory phenotype, as well as the dominance of the anti-inflammatory phenotype among all infiltrated macrophages.

Further, the effect of Oridonin on the phenotypic polarization of macrophages in the entire body was studied. Mononuclear cells (MNCs) derived from the spleens of EAN rats were determined with flow cytometry and results demonstrated a higher proportion of anti-inflammatory macrophages in spleen MNCs from the Oridonin treated EAN rats than in those of the control group rats (Figure 5E).

### Oridonin Inhibits Notch Signaling Protein Expressions in Sciatic Nerves of EAN Rats

As proved by the results from our *in vitro* study, Oridonin is a potent suppressor of the Notch signaling pathway and may thereby induce the phenotypic switch in macrophage polarization and suppress the inflammatory reactions. We therefore explored the expressions of the major products of the Notch pathway, including Jagged-2, Notch1, and Hes1 in the EAN sciatic nerves from both groups. Results from Western blotting analysis show potent local suppression of Jagged-2, Notch1, and Hes-1 by Oridonin treatment (Figure 5F). The involvement of the Notch pathway has been thereby approved in our EAN rats and all the representative molecules were modified following Oridonin treatments, in accordance with previous reports; therefore, no further investigations were performed to explore the pathway in detail as this has been previously reported in other studies.

## Discussion

As the prime animal model of human GBS and polyneuropathies, EAN is widely accepted and applied in disease mechanism investigation and development of novel therapeutic approaches. After we proved efficient anti-inflammatory activity of Oridonin and ability of inducing an anti-inflammatory phenotypic switch of macrophage polarization in cell culture, potential therapeutic values of Oridonin were further investigated in our animal model of human polyneuropathies and GBS. Our results show that either therapeutic or preventive treatments with Oridonin significantly shortened the disease duration, attenuated the peak severity, and paraparesis, or even delayed EAN onset. Oridonin treatments also significantly attenuated neuropathic pain, inflammatory infiltration of activated macrophages and other immune cells in the peripheral nerves and reduced microglial activation in the lumbar spinal cord. Interestingly, Oridonin treatment increased the systematic and local proportions of anti-inflammatory macrophages over the pro-inflammatory phenotypic population and resulted in the dominance of the anti-inflammatory phenotype among all infiltrated macrophages, which may be crucial for the anti-inflammatory activity, and ameliorated EAN progression. Our results from both *in vitro* and *in vivo* experiments suggest that the macrophage phenotypic switching effect of Oridonin may be associated with the inhibition of the Notch pathway.

As an effective diterpenoid isolated from *R. rubescens*, Oridonin has a variety of physiological and pharmacological properties/activities including anti-bacterial, anti-tumor, and anti-inflammatory. *R. rubescens* has a long history in TCM for the treatment of tumor and inflammatory diseases (Dong et al., 2014). The herb or its aqueous extract has been successfully applied in TCM for treatment of human inflammation such as gingivitis (Chen et al., 2009) and pharyngitis (Ma et al., 2011) for a long time. Several studies reported that Oridonin inhibited the expressions of COX-2 and iNOS by blocking NF-κB activity (Ikezoe et al., 2005; Leung et al., 2005); inhibited further pro-inflammatory molecules including IL-6, IL-2, IL-12, TNF-α, and IFN-γ; induced apoptosis of immune cells; as well as affected the anti-inflammatory target HO-1 (Hu et al., 2008); indicating its immunosuppressive and anti-inflammatory properties. However, a significantly modified expression of NF-κB was not observed in our EAN sciatic nerves, which indicated that other mechanisms which could be involved needed further investigation. Moreover, Oridonin has neuro-protective and anti-neuroinflammatory effects by modulating multiple microglial functions (Xu et al., 2009), suggesting its potential therapeutic application against inflammatory disorders of the nervous system.

In this present study, both preventive and therapeutic treatments with Oridonin significantly suppressed disease progression and eventually improved EAN outcome, by attenuating immune cell accumulation and reducing expressions of inflammatory cytokines in peripheral nerves, including the sciatic nerves and spinal roots. The infiltration of reactive leukocytes, mainly macrophages and T cells, into the PNS is the characteristic pathological change of EAN (Schabet et al., 1991); especially the activated macrophages triggered nerve demyelination by secretion of inflammatory mediators and direct phagocytic attack (Kiefer et al., 2001; Maurer et al., 2002). In EAN peripheral nerves, inflammatory cytokines are expressed and secreted by different immune cell types and modulate/regulate inflammatory responses. Pro-inflammatory molecules like IL-1β, IL-6, and iNOS are believed to promote EAN disease progression. IL-1β is considered a participant in the initiation of the autoimmune response of human neuropathies and the animal model EAN (Bettelli et al., 2008), IL-6 is believed to amplify local inflammation and be crucial for the EAN progression (Zhang et al., 2008b), iNOS produces pro-inflammatory NO (Abramson et al., 2001), and iNOS up-regulation in EAN was proved to contribute to the demyelination and even axonal damage in PNS (Conti et al., 2004). Therefore, Oridonin attenuated local inflammatory reactions and demyelination in the PNS and finally favored EAN outcome, by reducing local infiltration of reactivated immune cells and expression of inflammatory cytokines/molecules. In addition, the working concentrations used in our macrophage cell culture were comparable to the oral doses used in a previous study and presented significant dose-dependent anti-inflammatory patterns. The dosage was also comparable to orally administered Oridonin and the standard treatment followed in TCM (Xu et al., 2006).

Besides macrophages, we also observed a microglial accumulation in the lumbar spinal cord, together with pain hypersensitivity. While the EAN pathological changes were mainly studied and observed in the PNS, pathological changes in EAN spinal cord have drawn increased attention, especially focusing on spinal microglia (Piehl and Lidman, 2001; Nakamura, 2002), as they were considered to play a key role in inducing neuropathic pain in a variety of models (Moalem and Tracey, 2006). In the spinal cord of EAN animals, the activation of microglia is seen (Beiter et al., 2005), which is considered crucial in the induction of neuropathic pain (Raghavendra et al., 2003). Microglia are very sensitive to micro-environmental changes and spinal microglia are stimulated by various factors ranging from peripheral injury to central nervous system (CNS) inflammation. Reactive microglia in the spinal cord express various receptors and release inflammatory molecules acting either directly on pain transmitting dorsal horn neurons (nociceptive neurons) or on primary afferents, inducing raised sensitivity of the nociceptive neurons and contributing indirectly to central sensitization of neuropathic pain (Beggs et al., 2012; Ji et al., 2016). Therefore, activation of spinal microglia is essential and sufficient to initiate neuropathic pain. Suppression of spinal microglia can significantly relieve or even block the process of neuropathic pain in various animal models (Ji and Strichartz, 2004). Our study showed significantly reduced microglial accumulation, which might be due to attenuated stimuli from the periphery or direct inhibiting effects on spinal microglia. Our results indicate that Oridonin could suppress neuropathic pain, exert direct or indirect neuro-protective effects in the animal model, and possibly even in human patients, which makes the therapeutic and preventive effects of Oridonin more comprehensive.

As the major effector cells responsible for the main EAN pathological changes, macrophages’ activation states/status and phenotype may determine the disease progression. Based on our *in vitro* and *in vivo* results, the potent anti-inflammatory activity of Oridonin during EAN progression is, at least partially, due to the phenotypic switch toward anti-inflammatory macrophages and eventually their local dominance in peripheral nerves. This is also in accordance with previous studies demonstrating that anti-inflammatory activated macrophages played a protective and immune-modulatory role in EAN progression; as well as a switch of macrophage polarization from the pro-inflammatory phenotype toward the anti-inflammatory could resolve inflammation and thereby favor the outcome of different inflammatory disorders (Li et al., 2016; Klinkert et al., 2017); including animal models of EAN (Zhang Z. Y. et al., 2010; Han et al., 2016b). In our EAN rats, Oridonin raised the proportion of anti-inflammatory macrophages (ED2^+^ cells) substantially over the pro-inflammatory phenotype population not only locally in the peripheral nerves but also systemically as determined in the spleen, the most important storage organ of the peripheral immune cells. The latter may also suggest an improved environmental milieu in immune organs. Moreover, Oridonin efficiently reduced local expression of pro-inflammatory representative cytokines/molecules. Although the anti-inflammatory representative cytokine IL-10 was not significantly changed, considering the dramatically reduced number of total immune cells, a slight increase of IL-10 could also support the dominance of anti-inflammatory over pro-inflammatory cells.

Although the phenotypic switch of macrophage polarization has not been associated directly with Oridonin treatment, particularly in an inflammatory condition, previous data suggest possible involvement of the Notch pathway: the Notch pathway is a direct target of Oridonin and Notch inhibition could promote monocyte differentiation into anti-inflammatory macrophages (Singla et al., 2017; Xia et al., 2017). Dong et al. (2014) demonstrated that Oridonin could suppress the Notch activity, down-regulate Notch pathway proteins such as Jagged-2, Notch-1, and their downstream molecules, in the same manner as a Notch inhibitor could. Singla et al. reported that suppression of the Notch-1 pathway or of several further Notch signaling proteins decreased the inflammatory macrophages and expression of their cytokines, enhanced secretion of anti-inflammatory molecules, and subsequently promoted anti-inflammatory macrophage polarization (Singla et al., 2014), even for human primary monocytes (Singla et al., 2017). In the present study, we demonstrated that the anti-inflammatory switch of macrophage polarization in LPS-induced cell culture was promoted by Oridonin or the Notch inhibitor DAPT, but the transfection of Notch-1 could abolish this effect by Oridonin. This proves the effects of Oridonin on inhibition of the Notch-1 pathway. Further, expression levels of the Notch pathway proteins were also decreased locally in EAN sciatic nerves following Oridonin treatment, meanwhile proportions of anti-inflammatory macrophages were increased locally or systemically in the spleen. All these suggest that inhibition of the Notch pathway by Oridonin may be, at least partially, involved in the anti-inflammatory phenotypic switch of macrophage polarization.

## Conclusion

**FIGURE 6:**
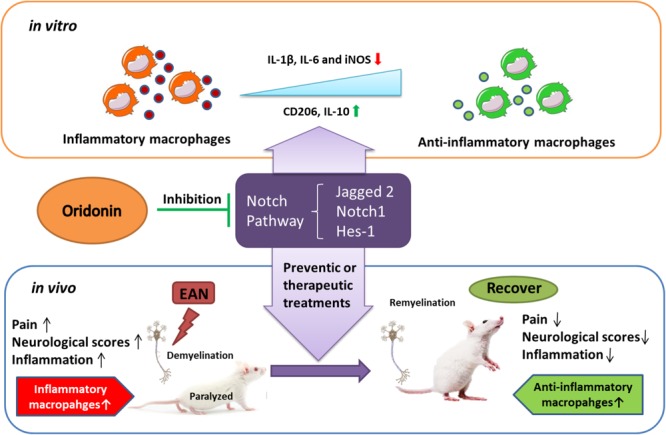
Schematic diagram showing how Oridonin ameliorates experimental autoimmune neuritis by promoting anti-inflammatory macrophages through blocking Notch pathway. Oridonin exhibits efficient anti-inflammatory activity and induced a switch in macrophage polarization to the anti-inflammatory phenotype through inhibition of the Notch pathway in our *in vitro* study. Further in the EAN rats, Oridonin significantly reduced neuropathic pain, demyelination, inflammatory cellular accumulations and inflammatory cytokines in peripheral nerves, effectively suppressed disease progression by attenuating local inflammatory reaction and increasing the proportion of immune regulating macrophages, possibly through blockage of the Notch pathway.

Oridonin ameliorated inflammatory EAN progression and finally favored the disease outcome through inducing the phenotypic switch of macrophage polarization toward the anti-inflammatory state, probably by the blockage of the Notch pathway (Figure 6). Therefore, with its proven safety and biocompatibility, Oridonin could be considered a potential anti-inflammatory therapeutic candidate of human GBS and other inflammatory neuropathies.

## Author Contributions

HS and Z-YZ designed the experiments and obtained resources and funding acquisition. LX and Z-YZ conducted the experiments with assistance from LL and C-YZ. LX, LL, C-YZ, and Z-YZ collected the data and contributed to the statistical analysis. LX, Z-YZ, and HS analyzed the data and wrote the manuscript. All authors read and approved the final manuscript.

## Conflict of Interest Statement

The authors declare that the research was conducted in the absence of any commercial or financial relationships that could be construed as a potential conflict of interest.

## Footnotes

1 flowjo.com

## Abbreviations

CFA: complete Freund’s adjuvant
CMC: carboxymethylcellulose
EAN: experimental autoimmune neuritis
FBS: fetal bovine serum
GBS: Guillain-Barré syndrome
GFAP: glial fibrillary acidic protein
HWT: hind-paw withdrawal threshold
IL-1β: interleukin-1β
iNOS: inducible nitric oxide synthase
IR: immunoreactivity
LFB: Luxol fast blue
LPS: lipopolysaccharide
MNCs: mononuclear cells
PNS: peripheral nervous system
SEM: standard errors of means
TBS: Tris-buffered saline
TNF: tumor necrosis factor
